# Glycyrrhiza polysaccharides inhibits PRRSV replication

**DOI:** 10.1186/s12985-023-02052-9

**Published:** 2023-07-05

**Authors:** Youbing Yang, Yongjian Liu, Ran Lou, Ying Lei, Gan Li, Zhiqian Xu, Xiangbin You

**Affiliations:** 1 College of Animal Science and Technology, Henan University of Science and Technology, Luoyang, 471023 China; 2 Luoyang Key Laboratory of Animal Genetics and Breeding, Luoyang, 471023 China

**Keywords:** Glycyrrhiza polysaccharide, PRRSV, CD163, SLA-7, NF-κB

## Abstract

Glycyrrhiza polysaccharide (GCP) is a natural plant active polysaccharide extracted from traditional Chinese medicine licorice. In this research, we studied the antiviral activity of glycyrrhiza polysaccharide against porcine reproductive and respiratory syndrome virus (PRRSV), a virus of the *Arteriviridae* family, with a high rate of variation and has caused huge economic losses to the pig industry in various countries since its discovery. Our results show that GCP can inhibit PRRSV replication in a dose-dependent manner. Furthermore, GCP could inhibit the mRNA expression of receptor genes CD163 and NF-κB p65 and promote the mRNA expression of the SLA-7 gene. Because of these results, GCP can be used as a candidate drug to prevent and treat PRRS.

**Supplementary Information:**

The online version contains supplementary material available at 10.1186/s12985-023-02052-9.

## Introduction

Porcine reproductive and respiratory syndrome (PRRS), commonly referred to as "blue ear disease", is a highly infectious disease of pigs caused by porcine reproductive and respiratory syndrome virus (PRRSV), mainly characterized by reproductive disorders and respiratory symptoms [1]. This disease can be transmitted vertically and horizontally. It can cause pregnant sows abortion, premature delivery, stillbirth, mummified fetus, and weak fetus, and induce a variety of respiratory diseases in piglets and growing pigs. This has brought severe economic losses to the national pig industry [2].

Presently, the prevention and control of this disease mainly rely on vaccination, and there are no effective drugs to treat it, which brings great challenges to the comprehensive prevention and control of PRRS [3]. However, in recent years, it has been found that traditional Chinese medicine has a good effect on the treatment of viral diseases, such as Houttuynia cordata [4], dandelion [5], and Polygonum cuspidatum [6]. Therefore, the development and research of traditional Chinese medicine preparation for treating this disease has a broad prospect but also provides a new experimental basis and ideas for treating other viral diseases.

Glycyrrhiza polysaccharide (GCP) is an active component extracted from glycyrrhiza. Its complex chemical structure makes it have many functions. It has been proven to enhance and regulate the body's immunity [7], be antibacterial [8], inhibit tumor growth [9], have antioxidant and other effects [10], and is often widely used as a feed additive in the aquaculture industry. In addition, it also has a strong antiviral effect and can significantly inhibit the invasion and proliferation of the virus. For example, GCP can significantly inhibit pseudorabies virus infection by interfering with virus adsorption and internalization [11]. However, the effectiveness of GCP against PRRSV infection is unknown.

In this study, we discussed the antiviral effect of GCP on PRRSV infection, and further explored its antiviral mechanism. Our data show that GCP has a good antiviral effect on the early stage of PRRSV infection, and GCP can potentially be used to treat PRRSV infection.

## Materials and methods

### Cell culture and glycyrrhiza polysaccharide (GCP) antiviral activitiy to PRRSV

Marc-145 cells (Honsun Biological, Shanghai, China) were diluted with 10% complete medium (DMEM1640) into 1 × 10^6^ cells/ml cell suspension. They were distributed into 12-well cell culture plates (Absin, Shanghai, China) in 1 ml per well. They were set as GCP (Lansealy, Luoyang, China) low, medium, and high concentration groups and control group, respectively, with 3 replicates in each group and incubated in a 5% CO_2_ cell incubator (HTFK, Beijing, China) at 37 ℃ for 24 h. When the cell density of the 12-well plate reached about 80%, the original cell culture medium was removed, the cells were rinsed twice with preheated sterile PBS (Servicebio, Wuhan, China), and 1 ml GCP with different concentrations (10, 20, 40 μg/ml) was added. In the control group 1 ml DMEM (Cytiva, Hangzhou, China) was added and incubated at 37 ℃ for 36 h with 5% CO_2_. Then the cells were washed with sterile PBS three times, and 1 ml of PRRSV was added at a concentration of 1 × 10^8^ PFU/ml (plaque forming unit/ml) to the cell culture and the virus titration method was MOI = 0.1 (multiplicity of infection = 0.1), and incubated at 37 ℃ for 1 h with 5% CO_2_.

### RNA extraction, cDNA synthesis, real-time fluorescence quantitative PCR

The culture medium was discarded, and the cells were washed three times with PBS. To each well, 0.5 ml Trizol (Absin, Shanghai, China) was added, and after 2 min, the cells were homogenized to lyse, then transferred to 1.5 ml RNA-free centrifuge tubes (JSHXRT, Jiangsu, China). The homogenate was maintained at room temperature for 5 min, and RNA was extracted according to the instructions of the Trizol kit. For the pig lung tissue sample, under ultra-low temperature in the presence of liquid nitrogen, the tissue sample was ground into a powder with a mortar, 0.2 g tissue sample was weighed as soon as possible, and 1 ml Trizol was added. The sample was homogenized and left for 5 min at room temperature to extract RNA.cDNA was synthesized using PrimeScript™ RT reagent Kit with gDNA Eraser (Takara, Beijing, China) reverse transcription kit. The first step was to remove genomic DNA contamination from total RNA. The reaction system was made up of 10 μl (5 × gDNA Eraser Buffer 2 μl, gDNA Eraser 1 μl, total RNA 1 μl, and ddH_2_O 6 μl). The preparation of the reaction solution was on ice. The reagents were gently mixed, and incubated at 42 ℃ for 2 min. The second step was to synthesize the cDNA, and the reaction volume was 20 μl (last reaction liquid 10 μl, 5 × PrimeScript® Buffer 4 μl, PrimeScript® RT Enzyme Mix 1 μl, RT Primer Mix 1 μl, ddH_2_O 4 μl). The prepared reaction solution was gently mixed, and incubated at 37 ℃ for 15 min, 85 ℃ for 5 s, and 4 ℃. The cDNA was stored at -20 ℃ for reserve.

Primers were designed based on sequences at NCBI as seen in Table 1. For the real-time fluorescent quantitative PCR test, SYBR® Premix Ex Taq™ II (Takara, Beijing, China) kit was used. The reaction volume was 20 μl (SYBR® Premix Ex TaqII 10 μl, PCR Forward Primer 1 μl, PCR Reverse Primer 1 μl, cDNA 1 μl, ddH_2_O 7 μl). The reaction was done by denaturation at 95 ℃ for 5 min, following 45 cycles (95 ℃ for 15 s, 60 ℃ for 30 s, 72 ℃ for 1 min), and ended with 72 ℃ for 10 min.

**Table 1:** Primers used for RT-qPCR

Primer name	Primer sequence (5’-3’)
ORF7-F	TAGGTGACTTAGGCACAGT
ORF7-R	TAAATATGCCAAATAACAAC
CD163-F	ATTCATCATCCTCGGACCCAT
CD163-R	CCCAGCACAACGACCACCT
SLA7-F	ACACGCATCTACAAGGACAC
SLA7-R	TGGTAGGTGTGAGACCCGGC
NF-κB P65-F	CATGCGCTTCCGCTACAAG
NF-κB P65-R	GGTCCCGCTTCTTTACACAC
U6-F	GCTTCGGCAGCACATATACT
U6-R	TTCACGAATTTGCGTGTCAT
β-actin-F	TCATCACCATTGGCATGAG
β-actin-R	AGCACTGTGTTGGCGTACAG

### Western blot

Cells were washed twice with PBS, and 125 μl RIPA (Servicebio, Wuhan, China) was added. After homogenizing for 5 min, the protein lysate was added to a 1.5 ml microtube and maintained in an ice bath for 30 min. After centrifugation at 12,000 g for 5 min, the supernatant was transferred to a new microtube. The protein was fractionated by SDS-PAGE (Solarbio, Beijing, China) and transferred to PVDF (Solarbio, Beijing, China) membrane by wet membrane transfer. The membrane was incubated with 5% skim milk powder was enclosed at room temperature for 2 h and incubated overnight at 4 ℃ with primary antibody to PRRSV nucleocapsid protein (Gene Tex-GTX129270, USA) diluted at 1:1000. The membrane was washed three times with TBST (1 L PBS + 5 ml Tween-20) to remove the unbound primary antibody, and then incubated with 1:500 diluted HRP labeled goat anti-rabbit IgG antibody (TransGen Biotech, Beijing, China) and slowly shake at room temperature for 1 h. After washing the membrane, the membrane was completely immersed in the ECL luminescent solution (Absin, Shanghai, China) and incubated for 1 min to color development and exposure. At the same time, β-tublin was used as the internal reference for the loading amount, and the image was taken directly by the protein glue imaging system (Omega Lum G).

### Immunofluorescence

Cell cultured in a 12-well plate were washed with PBS once. Then 0.1 ml of pre-cooled methanol was added to each well and fixed at 4 ℃ for 30 min, dried in the ventilator, washed three times with PBS, and dried. PRRS virus nucleocapsid protein antibody (Gene Tex-GTX129270, USA) diluted at 1:500 was added, 0.1 ml per well, and incubated at 37 ℃ for 30 min. After washing it three times with PBS, and it was dried, FITC-labeled Goat anti-Rabbit IgG antibody (TransGen Biotech, Beijing, China) in 1:100 dilution was added, 0.1 ml per well, and incubated in a 37 ℃ temperature box for 30 min away from light. The wells were washed three times with PBS, dried, and DAPI (Absin, Shanghai, China) dye solution was added at room temperature for 10 min, and slid the hole plate and use a fluorescence microscope to look at the image until it becomes clear.

### Weaner piglets were fed with different concentrations of GCP

A total of 75 healthy 28-day-old Duroc × Landrace × Yorkshire crossbred pigs with an initial body weight of (10.33 ± 0.55) were randomly assigned to 5 treatments with 5 replicates per treatment and 3 pigs per replicate (cycle) using a single-factor design. The pre-test period was 3 d and the formal period was 28 d. The experimental groups were fed GCP diets supplemented with 400 mg/kg, 800 mg/kg, 1200 mg/kg, and 1500 mg/kg, respectively, and the control group was fed the basal diet (Liuhe, Luoyang, China).

One piglet in each replicate of each treatment group was slaughtered, and lung tissue was collected and cut into small pieces with aseptic surgical clipping. The small pieces were placed in a cryopreservation tube, numbered, and quickly placed in liquid nitrogen (HUXI, Shanghai, China), and stored at −80 ℃ for testing. Primers were designed according to the mRNA sequences of CD163, SLA-7, and U6 genes published on NCBI. See Table 1 for specific primer information. Tissue-like RNA extraction, cDNA synthesis, and real-time fluorescence quantitative PCR are the same as the above methods.

### Statistical analysis

All experiments were performed at least three times in triplicate. Data were presented as mean ± SD. T-test was used for statistical analysis among different groups. Non-parametric Mann–Whitney Statistical test was used in vivo experiment due to the few number of animals available. A p-value of less than 0.05 was considered statistically significant, and a p-value of less than 0.01 was considered highly statistically significant.

## Result

### The GCP inhibits PRRSV replication

**Fig. 1:**
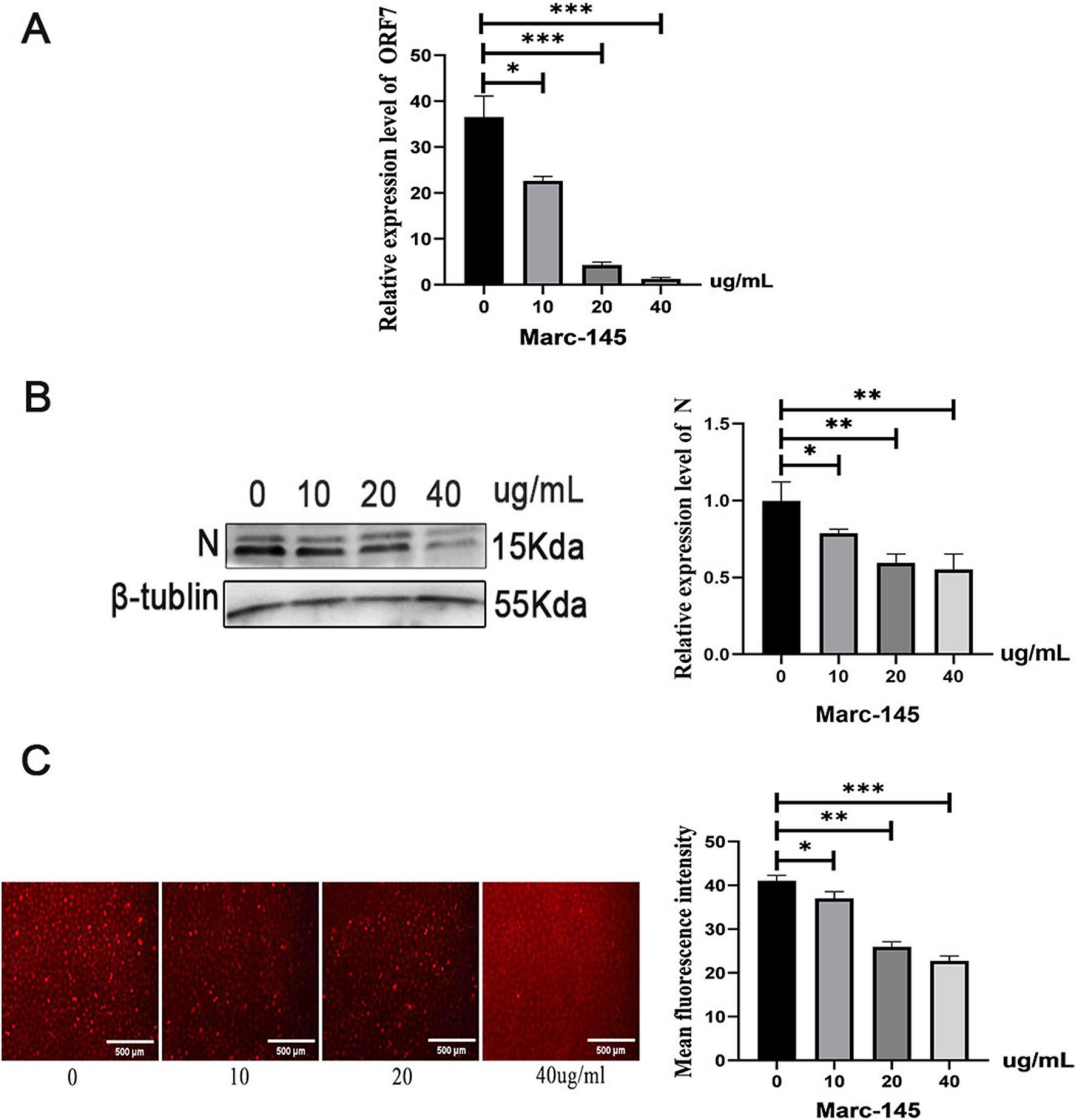
GCP inhibition PRRSV proliferation results. **A** Effect of GCP on PRRSV replication detected by qPCR. **B** Effect of GCP on PRRSV replication detected by WB. **C** Effect of GCP on PRRSV replication detected by immunofluorescence technique

To detect the effect of GCP on PRRSV replication, Marc-145 cells were treated with different concentrations of GCP, and then infected with PRRSV. The quantitative results show that with the increase in GCP concentration, the mRNA level of the ORF7 gene gradually decreases (Fig. 1A). The mRNA level of the ORF7 gene in the 10 μg/ml GCP treatment group was significantly lower than that in the control group (*P* < 0.05); however, the mRNA level of the ORF7 gene in the treatment groups with 20 μg/ml and 40 μg/ml GCP was extremely significantly lower than that in the control group (*P* < 0.01). Western blot results show that with the increase of GCP concentration, the virus protein content decreases continuously (Fig. 1B, Additional files 1, 2, 3). Compared with the control group, PRRSV N protein in the 10 μg/ml GCP treatment group decreased significantly (*P* < 0.05). Compared with the control group, PRRSV N protein in the treatment groups with 20 μg/ml and 40 μg/ml GCP shows a highly significant reduction (*P* < 0.01).

The effect of GCP on PRRSV replication was further detected by immunofluorescence. The results show that with the increase of GCP concentration, the red fluorescence brightness gradually decreases, indicating that the number of cells infected by PRRSV also decreases accordingly (Fig. 1C). Among them, the number of virus-infected cells in the 40 μg/ml GCP treatment group was the least.

### GCP inhibited the expression of CD163 and promoted the expression of SLA-7 in the Marc-145 cell

**Fig. 2:**
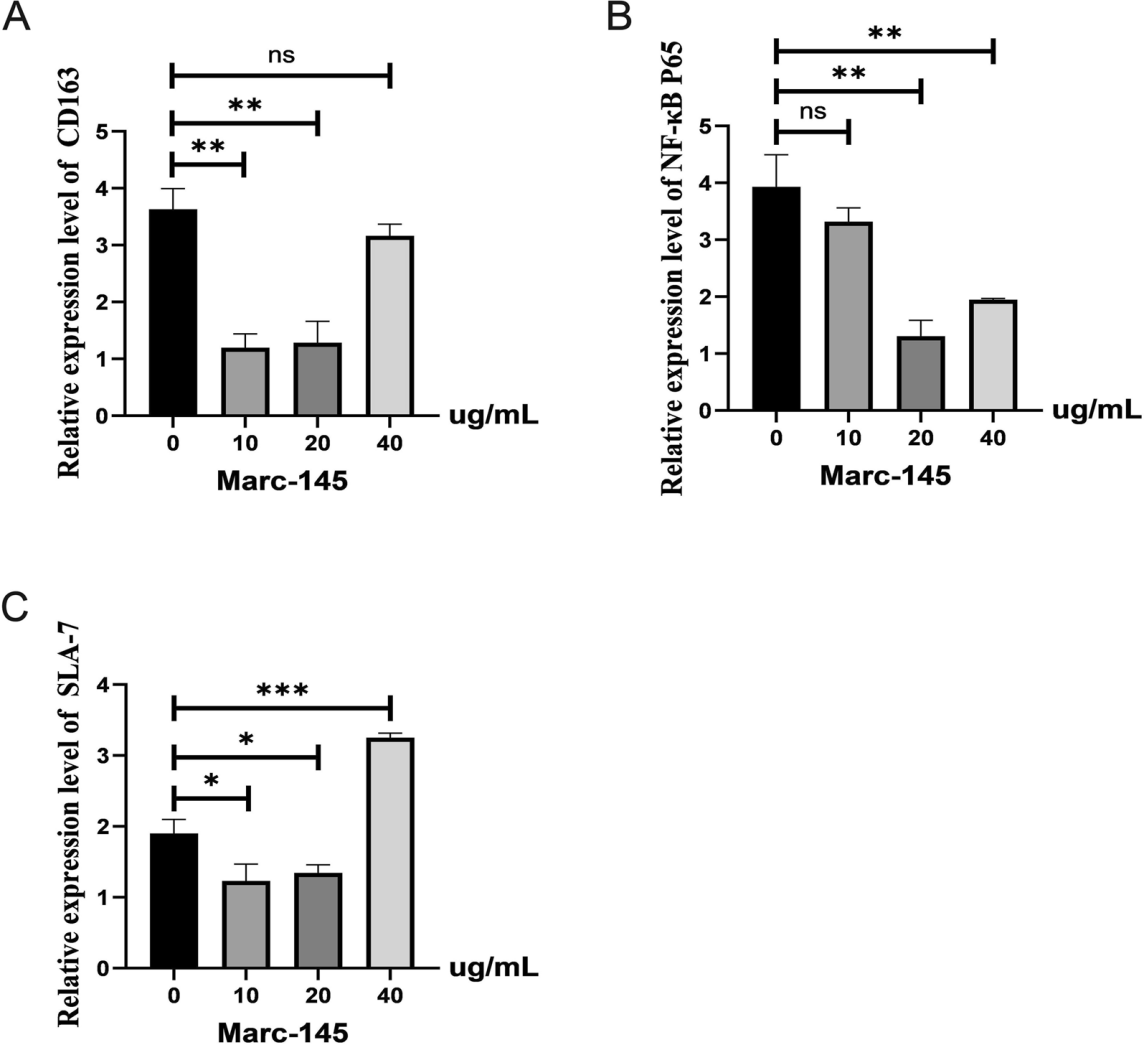
Effect of GCP on expression of related genes in Marc-145 cells. **A** Effect of GCP on CD163 gene mRNA expression in Marc-145 cells. **B** Effect of GCP on NF-κB P65 gene mRNA expression in Marc-145 cells. **C** Effect of GCP on SLA-7 gene mRNA expression in Marc-145 cells

CD163 is an important receptor molecule of the PRRSV invading host. We wanted to know whether GCP inhibited PRRSV replication by inhibiting the expression of CD163. As shown in Fig. 2A, in Marc-145 cells, the mRNA level of the CD163 gene in 10 μg/ml and 20 μg/ml GCP treatment groups were significantly decreased compared with the control group (*P* < 0.01). The mRNA level of the CD163 gene in the 40 μg/ml GCP treatment group was not significantly different from that in the control group (*P* > 0.05). In addition, the mRNA level of host innate immune-related genes has also changed. The results in Fig. 2B showed that the mRNA level of the NF-κB P65 gene in 20 μg/ml and 40 μg/ml GCP treatment groups were significantly lower than that in the control group (*P* < 0.01). There was no significant difference in the mRNA level of the NF-κB P65 gene in Marc-145 cells between the 10 μg/ml GCP treatment group and the control group (*P* > 0.05). As shown in Fig. 2C, the mRNA level of the SLA-7 gene in the 10 μg/ml and 20 μg/ml GCP treatment groups were significantly decreased compared with the control group in Marc-145 cells (*P* < 0.05). The mRNA level of the SLA-7 gene in the 40 μg/ml GCP treatment group was significantly higher than that in the control group (*P* < 0.01).

### GCP inhibited the expression of CD163 and promoted the expression of SLA-7 in piglets

**Fig. 3:**
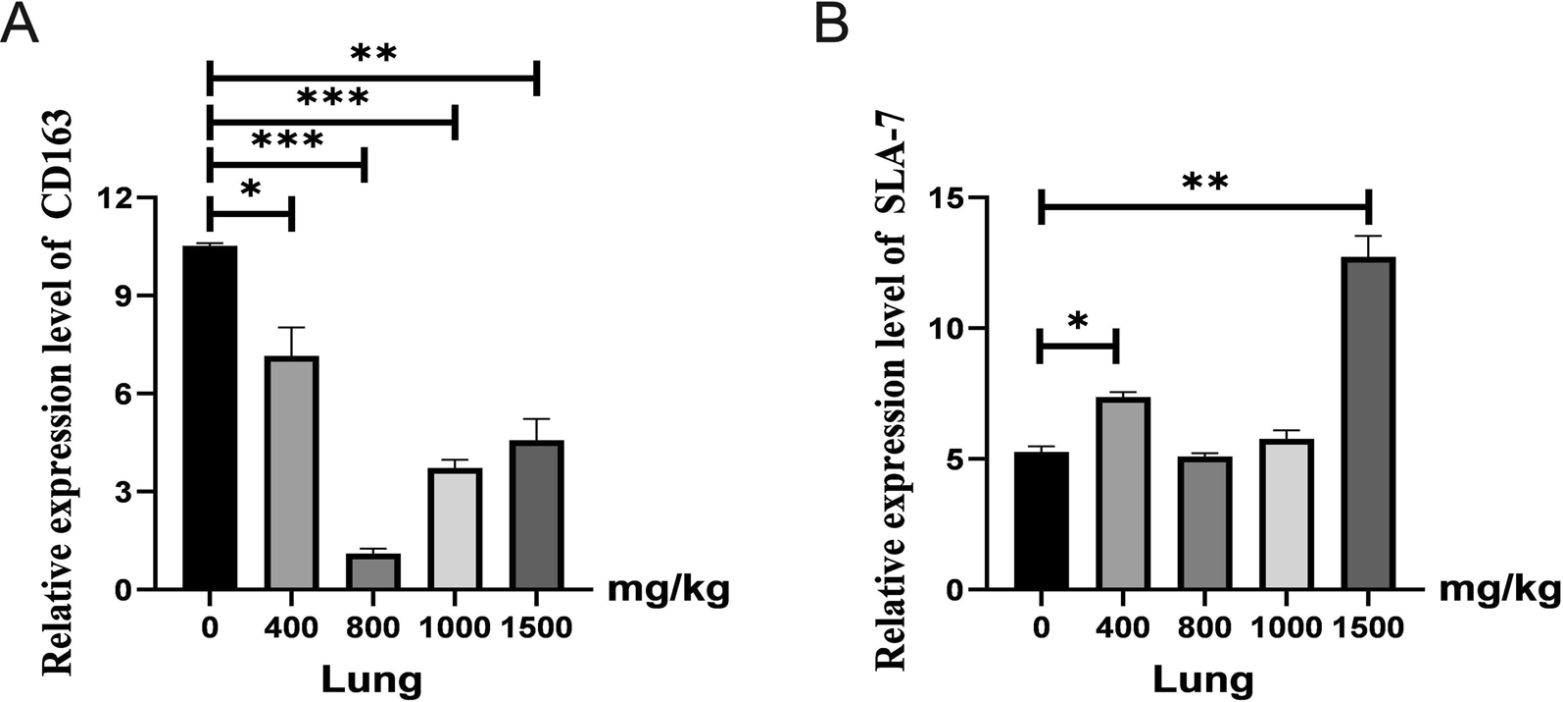
Effect of GCP on expression of related genes in the pig lung. **A** Effect of GCP on CD163 gene mRNA expression in lung. **B** Effect of GCP on SLA-7 gene mRNA expression in lung

To further verify the regulatory effect of GCP on host immune-related molecules, we used piglets fed with different concentrations of GCP to detect the mRNA level of CD163 in lung tissue. As shown in Fig. 3A, the mRNA level of the CD163 gene in the 400 mg/kg GCP treatment group was significantly decreased compared with the control group in pig lungs (*P* < 0.05). The mRNA level of the CD163 gene in 800 mg/kg, 1000 mg/kg, and 1500 mg/kg GCP treatment groups was significantly lower than that in the control group (*P* < 0.01), and the mRNA level was the lowest in 800 mg/kg. Similarly, as shown in Fig. 3B, the mRNA level of the SLA-7 gene in the 400 mg/kg GCP treatment group was significantly increased compared with the control group in pig lungs (*P* < 0.05). The mRNA level of the SLA-7 gene in the 1500 mg/kg GCP treatment group was significantly higher than that in the control group (*P* < 0.01).

## Discussion

PRRS is one of the most prevalent and notorious viral diseases, posing a sever threat to the survival of the global pig farming industry. The vaccine immunization is an important measure to prevent and control PRRSV. However, due to the high mutation and recombination evolution, PRRSV in pig farms has increasingly diversified, resulting in unsatisfactory prevention and control effects [12]. Consequently, it is essential to find new antiviral strategies and methods to resist its infection (Additional files 1, 2 and 3).

In recent years, with the deepening of research on Chinese herbal medicine, it has been found that it has the effects of promoting immunity and antiviral activity. Some researchers have found that the herbal extracts of traditional Chinese medicine contain molecules with significant antiviral activity at different stages of the PRRSV life cycle. These molecules achieve antiviral effects through different mechanisms [13]. This study,investigated the effects of GCP on PRRSV replication and expression of related genes.

ORF7 can encode the nucleocapsid protein (N protein) of PRRSV, and N protein shows the highest level in the PRRSV structural proteins and is also high in the viruses. Therefore, ORF7 is a vital target gene for virus detection, and the N protein is a crucial target protein for virus replication [14]. Zhenbang [15] detected the content of N protein expressed by the ORF7 gene and analyzed that PRRSV infection could be inhibited by UPR induction. Kaifeng [16] detected the expression of PRRSV N protein in PAM by Western Blot and found that overexpression of FAM134B in PRRSV-infected cells could inhibit PRRSV replication. If GCP is used to inhibit the expression of the N protein, it will prevent the assembly of PRRSV, thereby inhibiting PRRSV replication. This study investigated, the effect of GCP on PRRSV replication in Marc-145 cells. The results showed that adding different concentrations of GCP could significantly inhibit the mRNA level of ORF7 gene and significantly reduce the amount of translated protein. The effect of GCP on PRRSV was dose-dependent, and the best inhibitory effect was at 40 μg/ml. Duan [17] treated PRRSV-infected Marc-145 cells with glycyrrhizin extracted from glycyrrhizin root and found that the glycyrrhizin treatment significantly reduced PRRSV proliferation and PRRSV encoding N protein expression in a dose-dependent manner. The same conclusion was also obtained from glycyrrhiza polysaccharides extracted from licorice, which could significantly reduce the content of the N protein encoded by ORF7. Chaiwat [18] studied in vitro anti-PRRSV tests of extracts from seven medicinal plants (Sappanwood, Houttuynia cordata, etc.), and found that all seven extracts could inhibit PRRSV replication at high concentrations, and Sappanwood extract had the strongest inhibitory effect on PRRSV replication in Marc-145 cells.

The results also proved that GCP could significantly inhibit the replication of PRRSV. Studies have shown the use of curcumin in the treatment of virus-infected Marc-145 cells, and PAM experiments, it was found that curcumin can inhibit PRRSV infection by preventing viral internalization and virus-induced cell fusion. The GCP in this study also inhibited the infection of PRRSV, but the specific infection route needs to be further explored [19]. In the experiment of PRRSV replication induced by different concentrations of indigo woad root polysaccharides (10, 20, 40, 80 μg/ml), the results show that IRPS can well resist PRRSV replication in Marc-145 cells, mainly by affecting the adsorption of the virus to cells, thereby reducing the amount of viral RNA and protein synthesis in the infected cell. [20]. The results of this experiment were the same, with the increase of GCP concentration, the content of viral RNA gradually decreased. In conclusion, GCP can significantly inhibit PRRSV replication, but the specific action mechanism needs to be further studied. It is possible to prevent viral replication by blocking the attachment and entry of PRRSV into cells, or by inhibiting the replication and release of viral RNA or viral particle assembly through cytokine regulation, which needs further verification [21].

PRRSV has strict host specificity and is only susceptible to pigs, other species are not infected, and have a very specific cell tropism. It mainly infects porcine alveolar macrophages (PAM) in vivo and is only susceptible to Marc-145 and other cell lines in vitro. The reason is that there are specific receptors on the surface of these cells, mainly sialic acid adhesin receptor and caveator receptor B, among which CD163 is the main cellular receptor of PRRSV, mediating viral decapsidation and genome release during virus invasion [22]. Therefore, the mRNA expression level of the CD163 gene is closely related to PRRSV replication. Studies have reported that polysaccharides and their derivatives in the active antiviral ingredients of traditional Chinese medicine can hinder the adsorption process of viral particles on host cells from exerting antiviral effects, but the specific mechanism is still unclear. In this experiment, different concentrations of GCP were added to Marc-145 cells and piglet diet to investigate the effect of PRRSV receptor CD163 gene expression level. The results showed that the addition of 10 μg/ml and 20 μg/ml GCP in Marc-145 cells significantly decreased the mRNA expression level of the CD163 gene, and the mRNA expression level of the CD163 gene in pig lung was significantly decreased when different concentrations of GCP were added into the diet. Among them, 800 mg/kg showed the best inhibitory effect on PRRSV. Studies have shown that extracts of traditional Chinese medicine can prevent virus invasion by binding to the active site of CD163 and blocking the binding of viral structural proteins to host PAM cells, thus playing a role in the preventing and controlling PRRSV [23]. Whether the glycyrrhiza polysaccharides studied in this study can inhibit PRRSV replication in this way still needs to be further explored.

Porcine major histocompatibility complex (MHC), namely porcine leukocyte antigen (SLA), is a genetic region on the chromosome composed of closely linked and highly polymorphic gene loci. Its gene product is the MHC antigen, and its main function is antigen presentation, which is closely related to the disease resistance of animals [24]. Studies have shown that PRRSV infection down-regulates the expression of MHC Class II molecules (SLA-DR), leading to decreased antigen processing and presentation function and thus affecting the humoral immune response of the body to other pathogenic microorganisms [25]. Although many studies have been done on SLA at home and abroad, the anti-PRRSV effects of Chinese herbal medicine and polysaccharides on SLA family genes are rarely reported. This study showed that adding an appropriate concentration of GCP in Marc-145 cells and piglet diet can up-regulate the mRNA expression of the SLA-7 gene, and with the increase of dose, there is a gradual upward trend. These results indicate that GPS can enhance the host's immune function to a certain extent in vitro, play the role of anti-PRRV and protect the host from virus infection. The specific mechanism of action may be that GCP improves the antigen processing and presentation ability of Marc-145 cells and porcine alveolar macrophages, thus enhancing the host's humoral immune response and protecting the host from virus infection, which needs further experimental verification.

As a nuclear protein, NF-κB can specifically bind to target gene enhancer κB sequence, promote target gene transcription and expression, and participate in cell apoptosis, cell proliferation, differentiation, carcinogenesis and proliferation, immune reaction, and other biological processes. The NF-κB family consists of NF-κB l (p50), NF-κB 2 (p52), RelA (p65), RelB, and c-Rel, among which the p65 subunit plays an important role in innate and adaptive immunity [26]. Porcine reproductive and respiratory syndrome virus (PRRSV) infection has been shown to degrade IκB in Marc-145 cells and alveolar macrophages, thereby activating the NF-κB pathway. The specific mechanism of action is that the PRRSV nucleocapsid (N) protein activates the NF-κB pathway in Marc-145 cells, causing the virus to enter the nucleus to replicate and proliferate easily [27]. The results of this study showed that the mRNA expression of NF-κB p65 in 20 μg/ml and 40 μg/ml GCP treatment groups was significantly lower than that in the control group, and the expression level was the lowest at 20 μg/ml. This may indicate that GCP can down-regulate the mRNA expression of the NF-κB p65 gene, improve the immune capacity of the body and inhibit the proliferation of the virus. The mechanism may be that GCP reduces the phosphorylation and degradation of IκB, inhibits the activation of NF-κB in Marc-145 cells, and thus inhibits the nuclear transfer of the p65 subunit, thereby inhibiting the replication of PRRSV. Yulin [28] showed that the expression level of the IκBα mRNA was significantly decreased in the later PRRSV infection, leading to activation of the NF-κB pathway. Therefore, GCP may inhibit viral replication by blocking PRRSV-induced NF-κB. This requires further experimental verification.

This study showed that adding an appropriate concentration of glycyrrhiza polysaccharides to PRRSV-infected Marc-145 cells could inhibit the expression of the ORF7 gene and reduce the content of N protein in cells. The effect was dose-dependent, and the inhibitory effect on PRRSV was the best at 40 μg/ml. In addition, adding a certain concentration of GCP can increase the mRNA expression of the SLA-7 gene and improve the specific immunity of the body. At the same time, GCP can reduce the expression of the CD163 gene and NF-κB p65 gene in cells, inhibit the binding of virus and receptor, reduce the activation of NF-κB and block the nuclear transfer of p65 gene, thus inhibiting the proliferation of PRRSV. This study provides a theoretical basis for the application of Chinese herbal medicine in the prevention and treatment of PRRSV infection. It lays a corresponding theoretical basis for the research and development of anti-PRRSV drugs in the future.

## Supplementary Information

**Additional file 1**. The effects of different concentrations of GCP on PRRSV replication were detected by WB (Repeat 1).

**Additional file 2**. The effects of different concentrations of GCP on PRRSV replication were detected by WB (Repeat 2).

**Additional file 3**. The effects of different concentrations of GCP on PRRSV replication were detected by WB (Repeat 3).

## Data Availability

All relevant data and material is included in this publication.
