# Supplementary figures and images for: Glycyrrhiza polysaccharides inhibits PRRSV replication
Source: Virol J. 2023 Jul 5;20:140. doi: 10.1186/s12985-023-02052-9 (PMC10320881; doi:10.1186/s12985-023-02052-9)

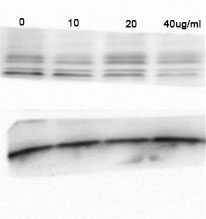

Supplement: Supplementary file 1 — Additional file 1. The effects of different concentrations of GCP on PRRSV replication were detected by WB (Repeat 1). [file 12985_2023_2052_MOESM1_ESM.jpg]

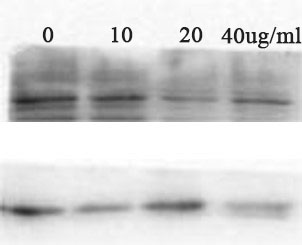

Supplement: Supplementary file 2 — Additional file 2. The effects of different concentrations of GCP on PRRSV replication were detected by WB (Repeat 2). [file 12985_2023_2052_MOESM2_ESM.jpg]

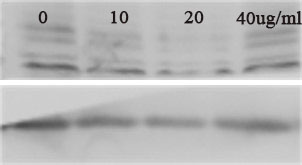

Supplement: Supplementary file 3 — Additional file 3. The effects of different concentrations of GCP on PRRSV replication were detected by WB (Repeat 3). [file 12985_2023_2052_MOESM3_ESM.jpg]
